# T9. CROSS-SECTIONAL ASSOCIATION OF MEMBRANE FATTY ACID COMPOSITION AND PSYCHOPATHOLOGY IN THE NEURAPRO-E STUDY

**DOI:** 10.1093/schbul/sby016.285

**Published:** 2018-04-01

**Authors:** Maximus Berger, Patrick D McGorry, Barnaby Nelson, Connie Markulev, Hok Pan Yuen, Miriam Schaefer, Nilufar Mossaheb, Monika Schlogelhofer, Stefan Smesny, Ian Hickie, Gregor Berger, Eric Chen, Lieuwe De Haan, Dorien Nieman, Merete Nordentoft, Anita Riecher-Rössler, Swapna Verma, Andrew Thompson, Alison Yung, G Paul Amminger

**Affiliations:** 1 James Cook University; 2 Orygen, The National Centre of Excellence in Youth Mental Health; 3 Orygen, The National Centre of Excellence in Youth Mental Health, The University of Melbourne; 4 Medical University of Vienna; 5 University Hospital Jena; 6 University of Sydney; 7 Child and Adolescent Psychiatric Service of the Canton of Zurich; 8 University of Hong Kong; 9 AMC-Academisch Psychiatrisch Centrum; 10 Academic Medical Center; 11 Mental Health Centre Copenhagen; 12 University of Basel Psychiatric Clinics; 13 Institute of Mental Health, Singapore; 14 University of Warwick; 15 University of Manchester

## Abstract

**Background:**

Converging evidence suggests that people at ultra-high risk (UHR) for psychosis have depleted levels of several fatty acids (FAs), and that changes in omega-3 (n-3) FA levels may indicate a higher risk for transition to psychosis. However, limited information is available on how FA deficiencies relate to psychopathology in individuals with UHR phenotypes. Here, we report the relationship between membrane FA levels and symptom severity in a study of individuals at UHR for psychosis.

**Methods:**

Data from 280 of 304 (92%) of participants of the NEURAPRO study, a multi-centre randomized-controlled trial of omega-3 fatty acids versus placebo, were used for the present analysis. All participants were aged between 13 and 40 years and met criteria for UHR for psychosis. Blood samples were collected at study baseline and month 6 (end-of-intervention). Membrane fatty acids were analysed using mass spectrometry as percentage of total fatty acids in erythrocytes. Pearson correlation coefficients were calculated between baseline erythrocyte fatty acid levels and scores on the Scale for the Assessment of Negative Symptoms (SANS) and Brief Psychiatric Rating Scale (BPRS).

**Results:**

Negative symptoms were positively correlated with one saturated FA (Tetracosanoic acid [24:0], R=0.272, p<0.0001), one n-3 FA (Eicosapentaenoic acid [20:5], R=0.142, p=0.017) and one n-9 FA (Nervonic acid [24:1], R=0.274, p<0.0001), and negatively correlated with one saturated FA (Palmitic acid [16:0], R=-0.224, p<0.0001), two n-6 FAs (Dihomo-y-linolenc acid [20:3], R=-0.201, p<0.001 and Linolelaidic acid [18:2], R=-0.333, p<0.0001), and one n-7 FA (Vaccenic acid [18:1], R=-0.172, p=0.004). BPRS scores were positively correlated with one saturated FA (Tetracosanoic acid [24:0], R=0.363, p<0.0001) and one n-9 fatty acid (Nervonic acid [24:1], R=-0.346, p<0.0001), and negatively correlated with two n-3 FAs (Dihomo-y-linolenc acid [20:3], R=-0.153, p=0.010 and Docosahexaenoic acid [22:6], R=-0.193, p<0.001), and two n-6 FAs (Arachidonic acid [20:4], R=-0.125, p=0.037 and Linoleic acid [18:2], R=-0.340, p<0.0001).

**Discussion:**

Consistent with a previous study, negative symptoms and general psychopathology were associated with levels of several classes of FAs in the present study. These findings support the relevance of membrane fatty acids for the onset of psychotic symptoms and indicate that FAs should be further evaluated as biomarkers in people at UHR for psychosis.

